# Emerging Adults’ Mental Health During the COVID-19 Pandemic: A Prospective Longitudinal Study on the Importance of Social Support

**DOI:** 10.1177/21676968211039979

**Published:** 2021-10-04

**Authors:** Yvonne H. M. van den Berg, William J. Burk, Antonius H. N. Cillessen, Karin Roelofs

**Affiliations:** 1 404818 Behavioural Science Institute, Radboud University, The Netherlands; 2 198328 Donders Institute for Brain, Cognition and Behaviour, Radboud University, The Netherlands

**Keywords:** COVID-19, mental health, social support, emerging adulthood, longitudinal

## Abstract

The aim of this longitudinal study was to investigate emerging adults’ mental health *before* and *during* the COVID-19 pandemic, and whether social support from mothers, fathers, and best friends moderated the *change* in mental health. Participants were 98 emerging adults (46% men) who were assessed prior to COVID-19 (*M*_age_ = 20.60 years) and during the first lockdown (*M*_age_ = 22.67 years). Results indicated that the pandemic did not uniformly lead to elevated levels of mental health problems, but instead depended on level of mental health problems prior to COVID-19 and the source of support. For emerging adults who already experienced more problems prior to COVID-19, more maternal support was related to *decreases* in general psychological distress and depressive symptoms, whereas more paternal support was related to *increases* in general psychological distress and depressive symptoms. Support from best friends were not associated with (changes in) mental health.

The lives of people across the world have been severely impacted by the COVID-19 pandemic not only in terms of major health risks and economic uncertainty but also in terms of increased emotional distress (Brooks et al., 2020). The COVID crisis has brought mental health challenges, particularly for emerging adults (Gruber et al., 2021). Emerging adulthood is considered a distinct developmental period, roughly from 18 to 25 years of age, characterized by increased opportunities for personal freedom and exploration (Arnett, 2000; Arnett et al., 2011). Although COVID-19 related restrictions impacted individuals of all ages, these restrictions may have especially impacted emerging adults’ opportunities for independence and personal and professional growth. Universities and colleges were closed, opportunities for vocational training or experience limited, and future employment opportunities uncertain. At the same time, opportunities to meet friends and close others were restricted. Social events (e.g., festivals, concerts, and parties) were canceled, and some had to return to living with parents after having lived independently. Thus, although the *physical* health impact of COVID-19 may be lower for emerging adults than for other age groups, they may be at particular risk for *mental* health problems. Therefore, the goal of the present prospective longitudinal study was to examine changes in emerging adults’ mental health prior to and during COVID-19, and whether support from close others (mothers, fathers, and best friends) moderated associations between mental health problems prior to COVID-19 and changes in mental health problems during the COVID-19 pandemic.

## Emerging Adults and COVID-19

The pandemic has impacted the mental health of young adults (e.g., Salari et al., 2020; Vindegaard & Benros, 2020; Wang et al., 2020; Xiong et al., 2020), and especially the health of emerging adults (Liu et al., 2020; Shanahan et al., 2020; Sun et al., 2020). Within 2 months after the US declared a state of emergency and the World Health Organization declared a global pandemic (April–May 2020), a substantial percentage of emerging adults reported clinically elevated levels of depression (43.3%), anxiety (45.4%), and PTSD symptoms (31.8%; Liu et al., 2020) that are higher than expected based on data for emerging adults before the pandemic (Liu et al., 2020). Similarly, young adults (age 21–31) and university students in China experienced high or even clinical levels of depression, anxiety, and traumatic stress symptoms during the first weeks of the COVID-19 outbreak (January–February 2020; Wang et al., 2020) and when they were under state-enforced quarantine (March–April 2020; Sun et al., 2020).

These cross-sectional studies provided important information about the prevalence of mental health problems. However, they do not indicate whether emerging adults’ mental health problems have changed during COVID-19. There have been a few prospective longitudinal studies on the consequences of the pandemic for adults’ mental health (Luchetti et al., 2020; Pierce et al., 2020; Planchuelo-Gómez et al., 2020), but the results of these studies have been somewhat inconsistent. For instance, significant deterioration of non-specific mental distress was found in the first month of the national lockdown (April 2020) in a nationwide sample of adults in the United Kingdom (April 2020), especially among emerging adults (age 18–24). Moreover, significant increases in mental health problems (e.g., anxiety, depression, and stress) were found during the strict confinement in Spain (March–May 2020; Planchuelo-Gómez et al., 2020). In contrast, no significant increase of loneliness was found during the acute phase of the pandemic (late March and late April 2020) in a nationwide sample of American adults (Luchetti et al., 2020).

There are also longitudinal studies of the consequence of COVID-19 for emerging adults, in particular; the results of these studies have also been inconsistent. An ecological momentary assessment study among 217 undergraduate students in America (Huckins et al., 2020) found increases in the number of anxious and depressive symptoms reported by students during the first weeks of the COVID-19 pandemic (March 2020) compared to the weeks before the pandemic (January–February 2020). A cohort study of 768 Swiss emerging adults also reported increased levels of distress (e.g., perceived stress and anger), but the effects were small and largely associated with pre-existing emotional distress and social stressors (e.g., being bullied, stressful life events, and social exclusion). That is, emerging adults who already experienced distress before the pandemic experienced more distress during the lockdown. These findings illustrate the need to disentangle pre-pandemic from pandemic-related factors and hence the need for longitudinal data on the changes in emerging adults’ mental health prior to and during COVID-19. In order to assess factors that contribute to vulnerability for or resilience against challenging life events such as the COVID-19 pandemic, it is also recommended to have data prior to the challenging situation that is not confounded by the acute effects of the event itself (Kalisch et al., 2017). Finally, identifying pre-pandemic risk factors is also relevant for policy guidelines and interventions for mental health during pandemics such as COVID-19.

## Impact of Social Support

Given the many changes that emerging adults experience and the additional challenges due to COVID-19, supportive relationships with close others may be of great importance. Social support can be defined as “emotional, informational, or practical assistance from significant others, such as family members, friends, or coworkers; support actually may be received from others or simply perceived to be available when needed” (Thoits, 2010, p. 46). Social support is thus a multifaceted construct that includes the degree to which people *receive* support, *feel* supported, and/or are in *need* for support. Irrespective of the crisis, there is clear evidence that social support is positively associated with physical and mental health (Asberg et al., 2008; Cohen, 2004). Experiencing closeness and emotional support is also a protective factor against the development of mental health problems. According to the stress-buffering model, social support buffers against the negative consequences of stressful life events as it helps individuals to better cope with negative circumstances (Cohen, 2004; Rueger et al., 2016; Schwarzer & Leppin, 1991). In other words, social support can protect people against the negative effects of stress and traumatic events on their mental and physical health. This is true across the lifespan, including the transitory and potentially challenging time of emerging adulthood (Arnett et al., 2014; Asberg et al., 2008; Galambos et al., 2006; Pettit et al., 2011), as well as in times of crisis (Grey et al., 2020; Norris & Elrod, 2006).

However, in a recent study by Szkody and colleagues (2020), social support did not act as a buffer between COVID-19 related stress and mental health problems. College students who worried about the effects of COVID-19 did not have fewer mental health problems when they felt more supported (e.g., perceived social support) or when they received more support (e.g., received social support). The limitations of social support render the COVID-19 crisis unique compared to other crises. The limited opportunities for human contact and social support (both giving and receiving) may cause frustration and disappointment (Norris & Kaniasty, 1996). This is in line with the social support deterioration deterrence model, which poses that the impact of a traumatic event or crisis on mental health can be through a disruption or deterioration of social support (Norris & Kaniasty, 1996; Norris et al., 2005). Indeed, Marroquín and colleagues (2020) showed that lack of contact opportunities due to personal distancing and stay-at-home orders was associated with increasing mental health problems, above and beyond individuals’ perceived social support.

However, the impact of social support on changes in emerging adults’ mental health during the pandemic may also depend on the source of support. Although emerging adults are gaining autonomy and the parent–child relationship becomes more egalitarian, parents continue to be a primary source of closeness and emotional support (Bartoszuk et al., 2019; Desjardins & Leadbeater, 2017; Galambos et al., 2018). In addition, maternal and paternal support are differentially associated with adjustment at this time (Desjardins & Leadbeater, 2017; Galambos et al., 2018). Similarly, support from friends may be distinctively related to mental health. Like parents, friends are an important source for comfort and reassurance in emerging adulthood (Collins et al., 2011). Yet, whereas some studies found that friend support buffered the impact of stress on mental health more than parent support (Lee & Goldstein, 2016), other studies found the opposite (Luecken & Gress, 2010; Meeus et al., 2007; Raffaelli et al., 2013). This also relates to emerging adults’ living situation, especially during COVID-19. Emerging adults who live with parents are not hindered in parental contact, but contact with friends is restricted. In contrast, for emerging adults who live with friends, friend support is not (or less) disrupted, while support from parents may be.

## Current Study

The aim of this study was to investigate changes in emerging adults’ mental health *before* and *during* the COVID-19 pandemic, and whether social support moderated the associations between mental health prior to COVID-19 and changes during COVID-19. We initially describe the COVID-19 situation of the sample at the time of assessment. This includes the national restrictions, physical and mental health, experienced stress and impact, and amount of support needed during COVID-19. Next, we examined whether support by parents (father and mother) and best friends were related to mental health problems prior to and during the crisis. Finally, we tested support from mothers, fathers, and friends as moderators of the association between mental health problems prior to COVID-19 and changes in mental health problems during COVID-19.

We formulated two opposing hypotheses. On the one hand, in line with the stress-buffering model, we hypothesized that social support would buffer against experiencing (more) mental health problems. That is, emerging adults with higher levels of social support were expected to experience *fewer* mental health problems both before and during the COVID-19 crisis, whereas emerging adults with lower levels of social support were expected to experience *more and increasing* mental health problems. On the other hand, in line with the social support deterioration deterrence model, we hypothesized that emerging adults with higher levels of social support would show *increasing* mental health problems during the COVID-19 pandemic, above and beyond pre-existing problems as a loss of contact may be more distressing when contact was of high quality. Finally, we explored whether the association between social support and mental health problems varied by source of support (e.g., father, mother, and friends).

## Method

### Recruitment and Participants

This study was part of the Nijmegen Longitudinal Study (NLS), an ongoing longitudinal study on social development in The Netherlands. This study started in 1998 with a community-based sample of 129 15-month-old infants and their primary caregiver (van Bakel & Riksen-Walraven, 2002). For this study, we used data of the 11th (February–September 2018; prior to COVID-19) and 12th Wave (May–June 2020; COVID-19). Data in Wave 11 were collected around the time of participants’ birthdates (as were all previous NLS assessments). In Wave 12, however, all participants were assessed in the same weeks during which the government-initiated restrictions due to the increasing number of COVID-19 infections. People were told to stay at least 1.5 m from others at all times, stay at home as much as possible, and have only one or two visitors at home per day. Public buildings (including schools and universities) were closed and social events were not allowed (e.g., festivals). Thus, the daily lives of our participants were severely disrupted. The pattern of results remained the same when taking into account the time lag between the dates of testing.

All participants who had not previously withdrawn their participation (*n* = 109) received an electronic invitation letter. If they were willing to participate, they received a link to an online questionnaire about various aspects of their social, emotional, and physical well-being. At the start of the online questionnaire, they were asked for informed consent. Procedures were approved by the Institutional Review Board of the Faculty of Social Sciences  at the Radboud University (2OU.007316).

In total, 98 participants completed measures at Wave 11 (*M*_age_ = 20.62 years, *SD* = 0.15; 49% men). Of these, 85 completed measures at Wave 12 (*M*_age_ = 22.67 years, *SD* = 0.20; 46% men). The majority (70%) received higher professional or university education. During the pandemic, 66% lived with housemates or romantic partners; 34% lived with parents.

### Measures

#### Demographic information

Participants indicated whether they identified as “Male,” “Female,” or “Other.” Age represented age at the day of assessment. Educational level was determined by asking what type of education they attended after secondary school. Answer options were grouped as *“None”* (No further education)*, “Secondary vocational education”* (MBO Level 1; MBO other), *“Higher professional education*” (HBO first year level; HBO Bachelor degree), and *“University education”* (University first year; University Bachelor degree; University Master degree).

#### COVID-19 health and exposure

Although emerging adults generally have a lower chance to become severely ill if infected by COVID-19, they may have pre-existing medical conditions that place them at risk. We therefore first asked participants whether they were at risk for severe or life-threatening symptoms of COVID-19 (0 = *No*, 1 = *Yes*, 9 = *I do not know*; DynaMORE, 2020). Next, we listed the main symptoms of COVID-19 and asked whether they were experiencing or had experienced them (0 = *No symptoms*, 1 = *A few symptoms*, 2 = *Symptoms, but tested negatively*, 3 = *Symptoms and awaiting test results*, 4 = *Tested positively*; COVGEN, 2020). In addition, we asked whether they were in mandatory quarantine (0 = *No*, 1 = *Yes*) or voluntary quarantine due to a confirmed or suspected infection (COVGEN, 2020). Finally, we wanted to know whether participants had been exposed to others infected by COVID-19. We therefore asked how many people they knew who had been infected and tested positively for COVID-19 (COVGEN, 2020).

#### COVID-19 perceived impact and stress

Four questions were used to assess perceived impact and stress due to the COVID-19 pandemic (COVGEN, 2020). First, participants rated on a visual analogue scale how much COVID-19 impacted their daily lives (i.e., “What is the general impact of the COVID-19 pandemic on your daily life?”; 0 = *No impact at all*, 100 = *A lot of impact*). Second, they indicated how much stress they experienced in the past 2 weeks due to COVID-19 (i.e., “Some people feel stressed because of the pandemic. We would like to know how much stress you currently experience and have experienced in the past 2 weeks due to the pandemic?”; 0 = *No stress at all*, 100 = *A lot of stress*). Third and fourth, they indicated whether their general stress level had changed due to the COVID-19 pandemic (−2 = *Worsened a lot*, 2 = *Improved a lot*) and their primary sources of stress (i.e., “What is currently your primary source of stress?” *“health concerns,” “financial concerns,” “impact on relationships with family members,” “impact on relationships with friends,” “impact on relationship with partner,” “general well-being due to quarantine or social distancing,” “other sources of stress, namely…”*).

#### COVID-19 general social support

Participants were asked about their general situation in terms of social support during the current COVID-19 pandemic. First, participants rated on a 100-point visual analogue scale “How much are you in need for social support from others*”* (0 = no need for support at all, 100 = very much in need of support). Second, to assess their means to gain social support if needed, they were asked “How do you try to receive support when needed?*”* Six answer options were offered *(“Phone calls,” “Video conversations (e.g., Skype, Zoom),” “Electronic communication (e.g., mail, text, app),” “personal contact,” “Social media (e.g., Facebook, Instagram, Snapchat),” “Other, namely …”*), of which they could chose as many or as few as they wanted (COVGEN, 2020). Third, they indicated whom they would turn to when in need for support by asking “From whom do you receive support when needed.” Ten answer options were given of which they could chose as many or as few options as they wanted (e.g., *“Partner,” “Mother,” “Father,” “Other family members (e.g., siblings, grandparents),” “Friends,” “Religious communities,” “Mental health care workers (e.g., psychologists, therapists),” “Medical health care workers (e.g., family doctor),” “Non-profit organizations,” “Other, namely …”*; COVGEN, 2020).

#### Social support (prior to COVID-19)

The Network of Relationships Inventory–Relationship Quality Version (NRI-RQV; Furman & Buhrmester, 1985) was used to assess participants’ perceived social support from father, mother, and best friend. First, participants indicated whether they had a mother or mother figure, father or father figure, and best friend. If so, they rated each of them on 15 items supportive relationship features (e.g., companionship, disclosure, emotional support, approval, and satisfaction) on a 5-point scale (1 = *Never/None*, 5 = *Almost always/The most*). An example item is “How satisfied are you with your relationship with your father/mother/name of best friend?” For each relationship, a mean score was computed, with higher scores indicating more perceived support (mothers: *M* = 3.33, *SD* = 0.70; fathers: *M* = 3.23, *SD* = 0.67; best friend: *M* = 3.91, *SD* = 0.51). Internal consistencies were good for all three relationships at both waves (Cronbach’s α = .90–.93).

#### Mental health (prior to and during COVID-19)

Participants rated their own mental health with the 90-item Symptom Check List-90-Revised (SCL-90-R; Arrindell & Ettema, 2003). Each item is a short description of a symptom (e.g., “headaches,” “feeling easily annoyed or irritated,” and “feeling fearful”). Participants rated the degree to which they experienced each symptom during the past week, including that day, on a 5-point scale (1 = *Not at all*, 5 = *A lot)*. A total score was computed, with higher scores reflecting more psychological distress (Cronbach’s α = .96 at both waves). A total score was also computed for each of eight subscales, with higher scores indicating more mental health problems. We present the results of potentially COVID-19 related mental health problems, namely anxiety (α = .84 and .86), depression (α = .88 and .93), and sleep difficulties (α = .62 and .75).

#### COVID-19 living situation

Participants were asked about their current living situation (*“I live with (one of) my parents,” “I live alone,” “I live with one or more housemates,” “I live with my partner,” “Other”*). They also indicated whether their current living situation had changed due to the COVID-19 pandemic (0 = *No*, 1 = *Yes*).

## Results

### Descriptive Statistics

Table 1 describes the demographic and COVID-19 related information. None of the participants indicated they had been infected; 62% reported not having any symptoms at the time of assessment. Ten participants were in quarantine due to a suspected or confirmed infection in their social contacts. The majority were not in a risk group due to pre-existing medical illnesses. Thus, the sample was healthy in terms of (risk for) COVID-19 infection.

**Table 1.** Demographic and COVID-19 Related Descriptive Information.

Educational Level	
Higher education	5.1% High school education
	24.4% Secondary vocational education
	31.6% Higher professional education
	38.7% University education
Living situation	
Current	34.1% Living with (one of my) parents
	8.2% Living alone
	44.7% Living with housemates
	8.2% Living with partner
	4.7% Other
Changed due to COVID-19?	83.5% No
	16.5% Yes
COVID-19 health situation	
Risk group	90.6% No
	4.7% Yes
	4.7% I don’t know
Exposure to COVID-19	63.1% 0 cases/unknown
	13.1% 1 case
	23.9% >1 cases
Own symptoms	62.7% No symptoms
	34.9% Mild symptoms
	2.4% Symptoms, but negative results or waiting for results
Quarantine	3.5% in mandatory quarantine
	8.2% in voluntary quarantine
COVID-19 impact and stress	
Impact on daily life	64.10 (*SD* = 21.69), range (10–100)
Stress	39.80 (*SD* = 25.03), range (0–100)
Change in stress	8.5% Worsened a lot
	48.8% Worsened a bit
	29.3% No change
	9.8% Improved a bit
	3.7% Improve a lot
Sources of stress	13.4% Health concerns
	29.3% Financial concerns
	45.1% Impact on relationship with family members
	22.0% Impact on relationship with friends
	17.1% Impact on relationship with partner
	34.1% General well-being due to social distancing or quarantine
	45.1% Other: Study/school
COVID-19 general social support	
Need for support	75.36 (*SD* = 19.53), range (15–100)
Means to gain support	60.7% Phone calls
	70.2% Video calls
	64.3% Electronic communication
	72.6% Personal contact
	41.7% social media
	4.8% Other (e.g., gaming/online platform)
Sources of support	46.4% Partner
	86.9% Mother
	77.4% Father
	58.3% Other family members
	94.0% Friends
	1.2% Religious community
	6.0% Mental health care professionals
	0% Medical health care professionals
	0% Non-profit organizations
	2.4% Other (e.g., colleagues)

The impact of COVID-19 on participants’ daily lives ranged from 10 to 100, with an average impact of 64.10 (*SD* = 21.69). Most experienced some degree of stress (*M* = 39.80, *SD* = 25.03, range 0–100). Still, 57.3% of the participants indicated that their stress level had worsened due to COVID-19. The most frequent sources of stress were concerns about impact on relationships with family members (45.1%), general well-being (34.1%), financial concerns (29.3%), and impact on relationships with friends (22.0%). Participants indicated that they were (very) much in need of social support (*M* = 75.36, *SD* = 19.53, range = 15–100). The majority indicated that personal contact was a way to receive support (73%), even though phone calls, social media, and online communication were also frequently mentioned. The primary sources of support were friends (94%), mothers (87%), and fathers (77%).

### Mental Health Before and During COVID-19

The means and standard deviations of the SCL-90 scores prior to and during COVID-19 as well as the average differences in SCL-90 scores are presented in Table 2. Mental health symptoms were, on average, not notably high according to the Dutch norm scores for the general population (e.g., norm group II; Arrindell & Ettema, 2003). Only 5.3% of the participants had clinically elevated levels of general psychological distress prior to COVID-19, and 12.0% reported elevated levels during COVID-19 (total score ≥ 183; Arrindell & Ettema, 2003). In the sample, the rates of clinically elevated levels of depressive symptoms were 9.5% prior to and 12.0% during COVID-19 (score ≥ 36); the rates of elevated levels of anxiety were 4.2% prior to and 7.2% during COVID-19 (score ≥ 22); and the rates of sleep difficulties were 12.6% prior to and 16.9% during COVID-19 (score ≥ 9; Arrindell & Ettema, 2003). Paired-sample *t*-tests to examine whether mental health problems (SCL-90 total and subscales) differed before and during COVID-19 yielded no statistically significant differences.

**Table 2.** Bivariate Associations Between Support From Mothers, Fathers, and Best Friends and Mental Health Problems Prior to COVID-19 and During COVID-19, and Changes in Mental Health Problems Before and During COVID-19.

			Mother support	Father support	Best friend support
Before COVID-19 (wave 11)	*M*	*SD*			
Total	125.93	28.96	−.19*	−.24*	.18
Anxiety	13.37	4.65	−.10	−.05	.21
Depression	23.68	7.69	−.12	−.27**	.16
Sleep	5.49	2.50	−.25*	−.12	−.02
*n*			95	91	66
During COVID-19 (wave 12)					
Total	131.49	34.59	−.15	−.14	.08
Anxiety	14.04	5.03	−.01	−.02	.06
Depression	25.49	10.25	−.18	−.20	.12
Sleep	5.69	2.74	.01	.12	−.09
*n*			83	78	57
Difference in symptoms					
Total	2.78	25.30	.01	.12	−.05
Anxiety	0.54	4.43	.08	.03	−.07
Depression	1.29	7.12	−.18	.02	.05
Sleep	0.12	2.71	.24*	.26*	−.01
*n*			82	78	57

*p < .05. ** *p* < .01.

A series of repeated measures ANOVAs examined whether changes in mental health problems differed for participants in three living situations: Living with parents before and during COVID-19 (*n* = 27), living with parents before COVID and living with peers during COVID-19 (*n* = 17), and living with peers before and during COVID-19 (*n* = 39). In these four analyses, the main effects of living situation and the interaction between living situation and time were all statistically non-significant. Thus, living situation was not related to mental health problems before and during COVID-19.

### Social Support and Mental Health

We initially examined whether support by parents (father and mother) and best friends were related to concurrent and prospective mental health problems. Table 2 presents correlations between relationship support with mothers, fathers, and best friends and the four measures of mental health problems before and during COVID-19, and changes in mental health problems between the two assessments.

Maternal and paternal support were negatively associated with SCL-90 total scores before COVID-19. Maternal support was negatively associated with sleep difficulties before COVID-19, and paternal support was negatively associated with depressive symptoms before COVID-19. So, higher levels of maternal and paternal support were concurrently associated with fewer total problems, higher levels of maternal support were related to fewer sleep difficulties, and higher levels of paternal support were associated with fewer depressive symptoms. The three support measures were not prospectively associated with mental health problems during COVID-19. Maternal and paternal support were positively associated with changes in sleep difficulties, indicating that higher levels of support were related to increases in sleep difficulties during COVID-19. Best friend support was not concurrently or prospectively associated with mental health problems before or during COVID-19.

Finally, we examined whether support by parents (father and mother) and best friends moderated the association between mental health problems before COVID-19 and changes in mental health problems during COVID-19. Table 3 presents the standardized regression weights from a series of multiple linear regression analyses that examined whether perceived support from mothers, fathers, and best friends moderated the association between mental health problems prior to COVID-19 and changes in problems during COVID-19 (i.e., the difference in problems before and during COVID-19). There were 13 participants who were missing an assessment of mental health problems during COVID-19. In order to account for these missing values, the regression analyses were performed with the lavaan package (Rosseel, 2012) in R (R Core Team, 2020) and employed full-information maximum likelihood to estimate the regression coefficients.

**Table 3.** Standardized Regression Weights for Perceived Support From Mothers, Fathers, and Friends and Mental Health Problems Prior to COVID-19 Explaining Changes in Mental Health Problems During COVID-19.

	Total	Anxiety	Depression	Sleep
	β	β	β	β
SCL wave 11	−.11	−.34*	−.01	−.46***
Mother support	−.12	.06	−.28*	.11
Father support	.12	−.05	.14	.15
Best friend support	.01	−.01	.03	−.09
SCL × mother	−.39**	−.16	−.54***	−.20
SCL × father	.44***	.25*	.55**	.11
SCL × friend	−.21	−.12	.08	−.11
R-square	.34	.17	.18	.30

*Note*. *n* = 98.

*p < .05. ** *p* < .01. *** *p* < .001.

For general psychological distress (e.g., total SCL-90 score), the interactions between mother support and problems before COVID-19 and between father support and problems were statistically significant. In order to interpret these interactions, we computed simple slopes for high (+1 *SD*) and low (−1 *SD*) levels of psychological distress prior to COVID-19. Maternal support was negatively associated with changes in distress for emerging adults with high levels of distress prior to COVID-19 (*b* = −14.094, *se* = 5.808, *p* = .017); maternal support was not associated with changes in distress for those with low levels of distress prior to COVID-19 (*b* = 5.888, *se* = 6.590, *p* = .374). Paternal support was positively associated with changes in distress for emerging adults with high levels of distress prior to COVID-19 (*b* = 15.360, *se* = 7.222, *p* = .036); paternal support was not related to changes in distress for those with low levels of distress prior to COVID-19 (*b* = −6.650, *se* = 7.057, *p* = .349). These follow-up analyses indicated that parental support was associated with changes in general psychological distress prior to and during COVID-19, but only for emerging adults who reported high levels of distress prior to COVID-19.

**Figure 1.**
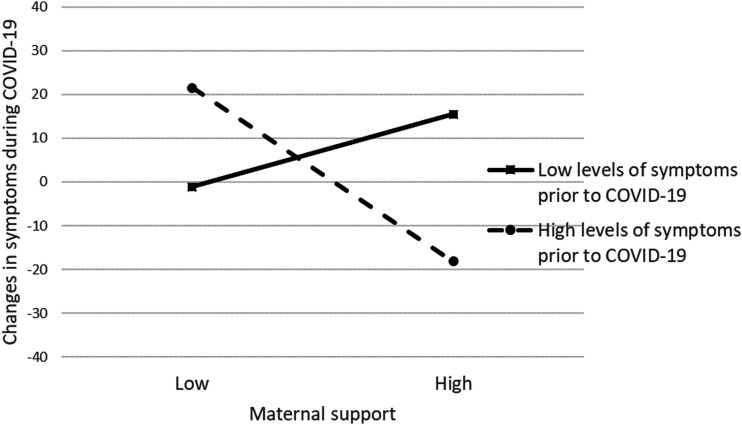
Simple slopes describing the association between maternal support and changes in symptoms during COVID-19 for emerging adults with low and high levels of symptoms prior to COVID-19.

**Figure 2.**
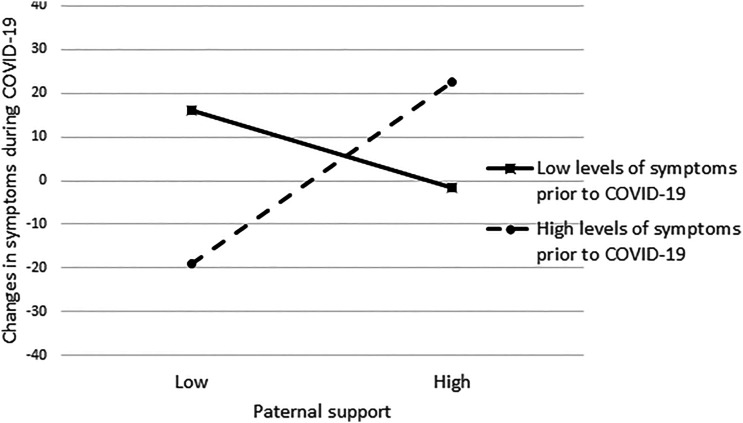
Simple slopes describing the association between paternal support and changes in symptoms during COVID-19 for emerging adults with low and high levels of symptoms prior to COVID-19.

Figures 1 and 2 present the plots of the simple slopes for maternal and paternal support, respectively. In Figure 1, the statistically significant slope describing emerging adults with high levels of distress indicates that low levels of maternal support were associated with increases in distress during COVID-19 (as indicated by positive values on the *y*-axis in the figure), and high levels of maternal support were associated with decreases in psychological distress during COVID-19 (as indicated by negative values on the *y*-axis of the figure). In Figure 2, the slope describing emerging adults with high levels of distress prior to COVID-19 indicates that low levels of paternal support were associated with decreases in their psychological distress during COVID-19, and high levels of support were associated with increases in distress during COVID-19.

For the three SCL-90 subscales, three of the nine interactions were statistically significant: between anxiety symptoms and father support, depressive symptoms and father support, and depressive symptoms and mother support. For each interaction, simple slopes were again computed for emerging adults with low and high levels of symptoms prior to COVID-19.

For anxiety symptoms, the simple slopes analyses indicated that paternal support was not associated with changes in anxiety during COVID-19 for emerging adults with high levels of anxiety symptoms before COVID-19 (*b* = 1.021, *se* = .897, *p* = .258), or for those with low levels of anxiety prior to COVID-19 (*b* = −1.703, *se* = 1.017, *p* = .098). For depressive symptoms, paternal support was positively associated with changes in depressive symptoms for emerging adults with high levels of depressive symptoms prior to COVID-19 (*b* = 5.545, *se* = 1.194, *p* < .001), but paternal support was not associated with changes in depressive symptoms for those with low levels of depressive symptoms prior to COVID-19 (*b* = −2.547, *se* = 1.343, *p* = .061). Maternal support was negatively associated with changes in depressive symptoms for emerging adults with high levels of depressive symptoms prior to COVID-19 (*b* = −6.886, *se* = 1.705, *p* < .001); maternal support was not associated with changes in depressive symptoms for those with low levels of distress prior to COVID-19 (*b* = 1.298, *se* = 1.601, *p* = .420). So, these follow-up analyses provided a pattern of results that was similar to those found for the general measure of psychological distress: maternal support was negatively associated with changes in depressive symptoms, and paternal support was positively associated with changes in depressive symptoms for emerging adults who were already experiencing high levels of symptoms prior to COVID-19.

## Discussion

The current study is among the first prospective longitudinal studies to investigate changes in emerging adults’ mental health prior to and during COVID-19. By accounting for pre-existing levels of mental health (prior to COVID-19), we demonstrated that the pandemic did not uniformly lead to increased levels of general psychological distress, depression, anxiety, or sleep difficulties. Specifically, maternal and paternal support were both associated with changes in psychological problems, but in opposite direction and only for emerging adults who already experienced problems prior to COVID-19. Surprisingly, supportive relationships with friends were not associated with (changes in) mental health problems in this sample.

Although the COVID-19 pandemic has affected people across the globe, research on its consequences for mental health is rapidly increasing (Salari et al., 2020; Vindegaard & Benros, 2020; Xiong et al., 2020). Even though reports of emerging adults’ well-being are still emerging, longitudinal studies are still scarce. Previous cross-sectional studies with large samples of emerging adults suggested high or even clinically elevated levels of mental health problems (Liu et al., 2020; Shanahan et al., 2020; Sun et al., 2020). In our data, the COVID-19 pandemic did not result in *heightened* levels of mental health problems. Some emerging adults reported clinical levels of psychological distress *during* the pandemic, yet similar numbers of emerging adults reported clinical levels of psychological distress *before* the pandemic. Our findings are in line with two other longitudinal studies of emerging adults’ emotional distress under COVID-19 (Luchetti et al., 2020; Shanahan et al., 2020), showing that COVID-19 did not necessarily increase psychological distress and that the strongest predictor of distress during the pandemic was pre-pandemic distress. This underscores the importance of further longitudinal studies of the unique consequences of COVID-19 for emerging adults. Moreover, it shows the importance of having data on the pre-COVID situation that is not confounded by the acute effects of COVID-19 itself, in order to assess factors that contribute to vulnerability for or resilience against challenging life events (Kalisch et al., 2017).

Moreover, investigations of the role of *both* maternal and paternal support (simultaneously) for emerging adults’ well-being are limited (Collins et al., 2011; Markiewicz et al., 2006). We demonstrated that social support from mothers and fathers was *uniquely* and *distinctively* related to changes in emerging adults’ mental health during COVID-19, especially for emerging adults who experienced mental health issues prior to COVID-19. Even though both mothers and fathers were mentioned as primary sources of support (87% and 77%, respectively), and their perceived levels of support were roughly equal, their impact on emerging adults’ mental health differed. Maternal support was related to *decreases* in general psychological distress and depressive symptoms for emerging adults who experienced higher levels of problems prior to COVID-19. Paternal support, on the other hand, was related to *increases* in general psychological distress and depressive symptoms for emerging adults who experienced higher levels of problems prior to COVID-19. Neither maternal nor paternal support was associated with changes in psychological problems for emerging adults who reported relatively fewer symptoms prior to COVID-19. These findings provide nuanced support for the stress-buffering hypothesis (Cohen, 2004; Rueger et al., 2016; Schwarzer & Leppin, 1991). A supportive relationship with mother seems to buffer against increased (COVID-19 related) mental health problems for emerging adults who already experienced problems prior to COVID-19, whereas paternal support seem to act as a risk for increased mental health problems for emerging adults who already experienced problems prior to COVID-19.

Although the distinct associations between paternal and maternal support with emerging adults’ mental health should be replicated, these initial findings contribute to the ongoing notion that emerging adults’ relationships with mothers and fathers differ in their function and significance (Desjardins & Leadbeater, 2011, 2017; Markiewicz et al., 2006). Previous studies have found that fathers are predominantly a source of instrumental forms of support (e.g., providing advice and active problem solving), whereas mothers are primarily a source of emotional support and care in emerging adulthood (Desjardins & Leadbeater, 2011, 2017). Moreover, maternal and paternal support seem to be differentially related to mental health and well-being (Desjardins & Leadbeater, 2011, 2017). In general, emotional supportive relationships with mothers have been found to be a better predictor of adjustment than supportive relationships with fathers (Desjardins & Leadbeater, 2017). One could thus argue that mothers and fathers provide complementary aspects of social support, which are uniquely and potentially additively associated with emerging adults’ well-being. Future studies should examine the unique and additive aspects of support from mothers and fathers to better understand their impact on emerging adults’ mental health and well-being.

Although friends—like parents—are an important source of intimacy and security (Markiewich et al., 2006), and most of our emerging adults (94%) indeed mentioned friends as a primary source of support, the quality of the relationship was not related to concurrent, prospective, or changes in mental health. This was unexpected, and we provide speculative explanations. One may be that parents and friends serve different purposes in emerging adulthood in general (Arnett, 2000), and these differences may be magnified in a crisis. Emerging adults turn to parents for advice and nurturance or a “safety net” (Carlson, 2014; Tanner, 2016); their friendships may be especially important in this time of crisis to socialize, be pleasantly distracted, discuss intimate topics (e.g., dating and sexuality), and form personal beliefs about COVID-19 (Barry et al., 2016; Carlson, 2014). In addition, emerging adults mentioned worries about school/work as well as the impact on family members as main sources of stress. It is likely that emerging adults turn to their parents rather than friends for advice and support on these topics (Carlson, 2014; Fingerman & Yahirun, 2016). A final possibility is that we assessed emerging adults’ experienced support from a single best friend. Even though this measure was just as reliable as the parental support measure, it might have been better to include a larger network of friends rather than one.

### Limitations

Despite the strengths and contributions of this paper, there are several caveats worthy of mentioning. First, the sample of 98 emerging adults was relatively small. Even though it enabled a more extensive assessment of social support from multiple close others, the sample size was too small to test interindividual as well as intraindividual differences. For instance, we were able to examine the unique contributions of mother, father, and friend, but not the impact of multiple supportive relationships at the same time (e.g., the interaction between maternal, paternal, and/or friend support). The relatively small sample size also did not allow us to more closely examine individuals who did not have a father (figure), mother (figure), or close friend during this crisis. In addition, we could not analyze the role of individual characteristics that are likely to exacerbate or diminish mental health problems during COVID-19, such as previous negative life events (Luecken & Gress, 2010; McLaughlin et al., 2010) and resilience (Li et al., 2021; Masten et al., 2006). Finally, there was not enough power to test the combination of intraindividual and interindividual differences, for instance, by examining variability due to gender composition of the parent-child dyad (Galambos & Kotylak, 2011).

Furthermore, we assessed the amount of support emerging adults experienced from parents and friends prior to COVID-19. We did not assess the degree to which they felt supported by each of them during the COVID-19 pandemic. At the same time, we did assess participants’ general need for support during the pandemic, but not whether they were more (or less) in need of support from parents or friends. In other words, our measures of social support prior to and during COVID-19 assessed different aspects (e.g., need vs. received) and sources of support (e.g., general vs. specific others). Consequently, we could not directly test whether emerging adults who were more in need of support and experienced a disruption in their previous support due to the COVID-19 pandemic would have (increasingly) more mental health problems than emerging adults who were less in need of support or did not experience a disruption in support. This information would have enabled a more stringent test of the social support deterioration deterrence model (Norris & Kaniasty, 1996; Norris et al., 2005). Moreover, studies indicate that the degree to which people feel supported (e.g., perceived support) and actual levels of received support are uniquely and distinctively associated with adjustment and well-being during stressful life events (Nurullah, 2012), including the COVID-19 pandemic (Szkody et al., 2020). Future studies on the importance of social support from close others for people’s mental health should therefore assess and contrast the different facets of social support (i.e., the degree to which people *receive* support, *feel* supported, and/or are in *need* for support).

Relatedly, our measure for social support was limited in terms of diversity in types of relationships as well as diversity in relationship qualities. Other relationships mentioned as (primary) sources of support were not examined, such as romantic partner or other family members (e.g., sibling and grandparents), whom emerging adults may also turn to for support in times of crisis (Feinberg et al., 2012; Scharf, 2016; Wetzel & Hank, 2020). Social support was also measured in terms of positive and supportive relationship features. Negative features (e.g., conflict) were not assessed.

Finally, while all measures of mental health prior to and during COVID-19 were reliable and comparable to other studies using the same questionnaire (Arrindell & Ettema, 2003; Smits et al., 2015), the internal consistency of the measure of sleep difficulties was somewhat lower; findings for this subscale should be interpreted more cautiously.

## Conclusion

Despite these limitations, this study contributes to our understanding of the impact of COVID-19 on emerging adults’ mental health. Our participants indicated that COVID-19 impacted their daily lives and was stressful to some degree, but did not (yet) result in notable increases of mental health problems. It is entirely possible that these effects will emerge later, as the pandemic endures and stress accumulates. Parental support was important; support from mothers was especially beneficial, while the absence of support from fathers was especially problematic. As the pandemic endures, continued research of the impact of this crisis on this generation of emerging adults is of the utmost importance. However, the implications of these findings may transcend the unique aspects of the COVID-19 pandemic. It shows that a global pandemic or crisis may affect everybody, but not necessarily to an equal degree. Moreover, it confirms that parents continue to be an important source of emotional support in emerging adulthood, not just when experiencing personal difficulties (Luecken & Gress, 2010; Meeus et al., 2007; Raffaelli et al., 2013) but when it concerns a nationwide or even global crisis. Finally, it shows that emerging adults may be particularly vulnerable for the development of mental health problems if they are restricted in their social contact as it limits their opportunities for personal and professional growth as well as independence.
